# Genomic representation predicts an asymptotic host adaptation of bat coronaviruses using deep learning

**DOI:** 10.3389/fmicb.2023.1157608

**Published:** 2023-05-05

**Authors:** Jing Li, Fengjuan Tian, Sen Zhang, Shun-Shuai Liu, Xiao-Ping Kang, Ya-Dan Li, Jun-Qing Wei, Wei Lin, Zhongyi Lei, Ye Feng, Jia-Fu Jiang, Tao Jiang, Yigang Tong

**Affiliations:** ^1^State Key Laboratory of Pathogen and Biosecurity, Beijing Institute of Microbiology and Epidemiology, AMMS, Beijing, China; ^2^Beijing Advanced Innovation Center for Soft Matter Science and Engineering (BAIC-SM), College of Life Science and Technology, Beijing University of Chemical Technology, Beijing, China

**Keywords:** bat coronavirus, asymptotic adaptation, deep learning, dinucleotide composition representation (DCR), convolutional neural networks

## Abstract

**Introduction:**

Coronaviruses (CoVs) are naturally found in bats and can occasionally cause infection and transmission in humans and other mammals. Our study aimed to build a deep learning (DL) method to predict the adaptation of bat CoVs to other mammals.

**Methods:**

The CoV genome was represented with a method of dinucleotide composition representation (DCR) for the two main viral genes, *ORF1ab* and *Spike*. DCR features were first analyzed for their distribution among adaptive hosts and then trained with a DL classifier of convolutional neural networks (CNN) to predict the adaptation of bat CoVs.

**Results and discussion:**

The results demonstrated inter-host separation and intra-host clustering of DCR-represented CoVs for six host types: Artiodactyla, Carnivora, Chiroptera, Primates, Rodentia/Lagomorpha, and Suiformes. The DCR-based CNN with five host labels (without Chiroptera) predicted a dominant adaptation of bat CoVs to Artiodactyla hosts, then to Carnivora and Rodentia/Lagomorpha mammals, and later to primates. Moreover, a linear asymptotic adaptation of all CoVs (except Suiformes) from Artiodactyla to Carnivora and Rodentia/Lagomorpha and then to Primates indicates an asymptotic bats-other mammals-human adaptation.

**Conclusion:**

Genomic dinucleotides represented as DCR indicate a host-specific separation, and clustering predicts a linear asymptotic adaptation shift of bat CoVs from other mammals to humans via deep learning.

## 1. Introduction

RNA viruses from natural reservoir hosts continuously pose a threat to human health, such as coronaviruses (CoVs) from bats (Liu et al., 2021; Wang et al., 2022) and avian influenza viruses from birds (Liu et al., 2014; Sun et al., 2014; Deng et al., 2017). In particular, bat-originated CoVs either caused high-pathogenic but low-transmissible infections of Severe Acute Respiratory Syndrome (SARS) or Middle East Respiratory Syndrome (MERS) or launched a widespread pandemic of low-pathogenic human CoVs, such as HcoV-NL63, HcoV-229E, HcoV-OC43, and HKU1 (Su et al., 2016; Forni et al., 2017). The ongoing global spread of SARS-CoV-2 has not only caused huge damage to public health (WHO, 2022) but also radically changed social habits and lifestyles (West et al., 2020; El-Sayed and Kamel, 2021). Orthocoronavirinae, known as CoV, is one of the two subfamilies in Coronavirinae and consists of four genera of alpha-, beta-, gamma-, and delta-coronaviruses that infect mammalian or avian hosts, especially those specific to species (Woo et al., 2012). The two former CoV members only infect mammals, and the two latter CoVs dominantly infect birds, with some exceptions for mammalian infection (Woo et al., 2012; Ji et al., 2022). According to current CoV databases, almost all human CoVs, with HcoV-OC43 and HKU1 as the exceptional origins of rodents (Forni et al., 2017), have been indicated to have originated in bats (Cui et al., 2019; Ruiz-Aravena et al., 2022). SARS-CoV-2 likely originated from bats as well (Zhou et al., 2020). Additionally, approximately half of the 20 Alphacoronavirus or Betacoronavirus species were identified only in bats (Cui et al., 2019). Taken together, bats are most likely natural reservoirs of CoVs.

Bats are the second largest order of mammals after rodents, widely inhabiting all continents except Antarctica (Gentles et al., 2020), accounting for approximately one-third of CoV sequences before Coronavirus Disease 2019 (COVID-19) (Ruiz-Aravena et al., 2022). The natural reservoir role of bats for CoVs is attributed to host/virus co-existence in an equilibrium pattern, which is also interpreted as the virus adapting to the host, enabling effective bat infection and inter-bat transmission but with limited pathogenicity (Li et al., 2020, 2022) due to several factors. First, bats exhibit extraordinary immune tolerance, which maintains a moderate immune response to invading viruses such as CoVs, leading to limited viral replication and asymptomatic or mild CoV infections (Baker et al., 2013; Olival et al., 2017; Banerjee et al., 2018; Skirmuntt et al., 2020; Sia et al., 2022). Second, bats have a high body temperature, which resembles other mammals' febrile responses and infection immune responses, helping to keep virus infections at a tolerable level (O'Shea et al., 2014). Third, factors such as the large and closely aggregated population, sustained flight capability, and extreme roosting proximity of bats support the widespread and sustained existence of CoVs within the bat population (Maganga et al., 2014; Olival et al., 2017; Roes, 2020). Thus, the sustained infection and transmission in bats provide CoVs with a high probability of accumulating mutations, leading to variants with marginal adaptation to other mammalian hosts and causing spillover infections in humans and other mammals.

Numerous bat CoVs have been isolated and sequenced in recent years. A total of 78% (2,209/2,820) of the recorded CoV sequences in NCBI were uploaded since 2015, before the COVID-19 pandemic (https://www.ncbi.nlm.nih.gov/nuccore). However, it is challenging to assess the risk of new bat CoV isolates that cause infection or pandemics in human or other mammalian populations (Seyran et al., 2021). Traditional phylogenetic analysis can sufficiently evaluate the cross-species infection risk or any bat CoV (Lima et al., 2013; Seyran et al., 2021). More recently, machine learning or deep learning approaches based on big sequencing data have led to remarkable predictions of the host adaptation (Li et al., 2022; Nan et al., 2022), evolution (Hie et al., 2021), transmissibility (Fischhoff et al., 2021), virus–host interaction (Dey et al., 2020), and pathogenicity (Gussow et al., 2020) of SARS-CoV-2 and other viruses (Li et al., 2020). Host-specific compositional features in the virus genome have been indicated by the representation traits, such as dinucleotides (DNTs) (Li et al., 2020), DNT composition representation (DCR) (Li et al., 2022), and Uniform Manifold Approximation and Projection (UMAP) (Hie et al., 2021). Unfortunately, there is no pipeline or framework available to predict the adaptation of recorded or newly isolated bat CoVs to main mammalian hosts, such as Primates, Rodents, Artiodactyla, Suiformes, or Carnivora.

The present study aimed to represent the genome composition of the two main genes, i.e., *Spike*, the receptor binding glycoprotein, and *ORF1ab*, the RNA-dependent RNA polymerase complex, and then to predict the adaptive host of recorded or newly isolated CoVs. In this study, the viral genome representation and adaptive host prediction framework provide an intelligent approach to assessing the risk of cross-species infection and transmission for bat CoVs.

## 2. Methods

### 2.1. Data preprocessing and genomic compositional trait parsing of ssRNA viruses

Full genome sequences of coronaviruses were downloaded from the NCBI nucleotide database (https://www.ncbi.nlm.nih.gov/nuccore) and cleaned by removing records with multiple imprecise nucleotides or filtering with sequence length thresholds of 27,000 and 32,000 bp. Six types of adaptive host labels, including Chiroptera (CHI), Artiodactyla (ART), Suiformes (SUI), Rodents/Lagomorphs (Rodents, ROD), Carnivora (CAR), and Primates (PRI), were extracted from the “FEATURES”-“source”-“host” of each sequence record in the genebank sequence files and were manually checked one by one according to the host family (genus for porcine CoVs). The coding sequences of the two main CoV genes, *ORF1ab* and *Spike*, were parsed with the Biopython Python package. Genomic nucleotide composition traits of mononucleotide (NT), dinucleotide (DNT), DNT composition representation (DCR), trinucleotide (codon), codon pair, and amino acid (AA) were counted as a frequency value for each *ORF1ab* or *spike* sequence sample with a nucleotide counting script. The traits of NT, DNT, and DCR were counted as codon nucleotide-dependent sequences (Li et al., 2022). In sum, 12, 48, 1,536, 64, 3,721, and 20 features of the aforementioned six types of compositional traits were counted and utilized for genome composition analysis.

### 2.2. Clustering in genomic composition traits of coronaviruses

To visualize data distribution and clustering, dimension reduction was performed using Principal Component Analysis (PCA) and t-Distributed Stochastic Neighbor Embedding (t-SNE) for the full-dimension features of 12 NTs, 48 DNTs, 1,536 DCRs, 64 codons, 3,721 codon pairs, or 20 Aas for *ORF1ab* or *Spike*. PCA and t-SNE were performed using sklearn.decomposition.PCA (Jolliffe and Cadima, 2016) and sklearn.manifold.TSNE (https://scikit-learn.org/stable/about.html#citing-scikit-learn), respectively. Two main components (PCA1 and PCA2, or t-SNE1 and t-SNE2) were plotted with a host label for each data point using the Python Seaborn package. An unsupervised machine learning approach based on hierarchical clustering was used to observe the clustering and homology of CoVs with various adaptation host labels based on full-dimension features of each compositional trait. Euclidean distance was utilized as a hierarchical clustering scalar, and hierarchical clustering was performed using the sns.clustermap package. Additionally, to balance the biased sample number of CoVs with the six host labels, random down- and up-sampling were performed using the imblearn.over_sampling.SMOTE package before dimension reduction and visualization.

### 2.3. Building and training of deep learning predictors for adaptive hosts

To predict the adaptation of bat CoVs to other mammalian hosts, a deep learning predictor of convolutional neural networks (CNN) (Li et al., 2022) was built based on the 1,536 DCR features and five host labels (ART, SUI, ROD, CAR, and PRI). Five adaptive hosts were labeled as {0: 'SUI,' 1:'ART', 2:'ROD', 3:'CAR',4: 'PRI'}, respectively. Two packages, pandas.DataFrame.sample and imblearn.over_sampling.SMOTE, were utilized to perform down- and up-sampling to maintain the sample number balance of various host-originated CoVs. Two CNN models were built, one for *ORF1ab* and another for *Spike*. sklearn.model_selection import train_test_split was utilized for random training/test data splitting with a test data size of 25%. All bat CoV samples, either for *ORF1ab* or *Spike*, were not included in either the training or test data sets to avoid data leaks and were only utilized for the adaptive host prediction with trained models. The 1,536 DCR features of *ORF1ab* or *Spike* sequences were reshaped into an array of (6, 16, 16) for a 3D-CNN model of three convolutional layers. Out-channels of (8, 16, 32), a stride of (1, 1, 1), a padding of (0,1,1), and a kernel_size of (1, 3, 3) were set for the three layers of CNN. ReLU activation and average pooling were followed for each CNN layer. Two linear transformations were performed into the 192- and 5-dimensions, respectively, from the 768- and 192-dimensions of a fully connected layer. The sigmoid activation function was utilized for the 192-dimensions of the full-connected layer after one time of linear transformation to output prediction, and the Softmax function was utilized to output the prediction probability. A learning rate of 0.001 and a training epoch of 50 were set uniformly for the *ORF1ab* or *Spike* 3D-CNN model.

### 2.4. Evaluating the deep learning predictor

To evaluate the predictor's performance, the prediction of adaptive hosts and the adaptation probability for each host label were the outputs for each model. The confusion matrix (Townsend, 1971) and micro-average receiver operating characteristic (ROC) (Fawcett, 2005) with AUCs were plotted. A pair plot of the PCA-reduced, fully connected layer data (768-dimension) was performed with the two components to visualize the separation or clustering of the CoVs from different or the same host(s). The PCA1 value was also plotted and compared between/among these CoVs. Statistical significance in the PCA1 value of the PCA-reduced fully connected data was analyzed using an unpaired, non-parametric Mann–Whitney test, based on the hypothesis of non-Gaussian data distribution using GraphPad Prism 9.

### 2.5. Predict adaptive hosts for bat coronaviruses via the deep learning predictor

To evaluate the adaptive host(s) of bat CoVs, each of the bat CoV samples was predicted using the trained *ORF1ab* or *Spike* 3D-CNN model based on 1,536 *ORF1ab* or *Spike* DCR data. The adaptation and adaptation probability were output for each of the five hosts (ART, SUI, ROD, CAR, and PRI). The probability vector (five probability values) of all bat CoVs and the CoVs from other mammalian hosts were reduced to two main values by PCA and plotted, with each data point labeled with its host type or virus name.

## 3. Results

### 3.1. The architecture of genomic parsing and adaptation for predicting bat CoVs

**Figure 1:**
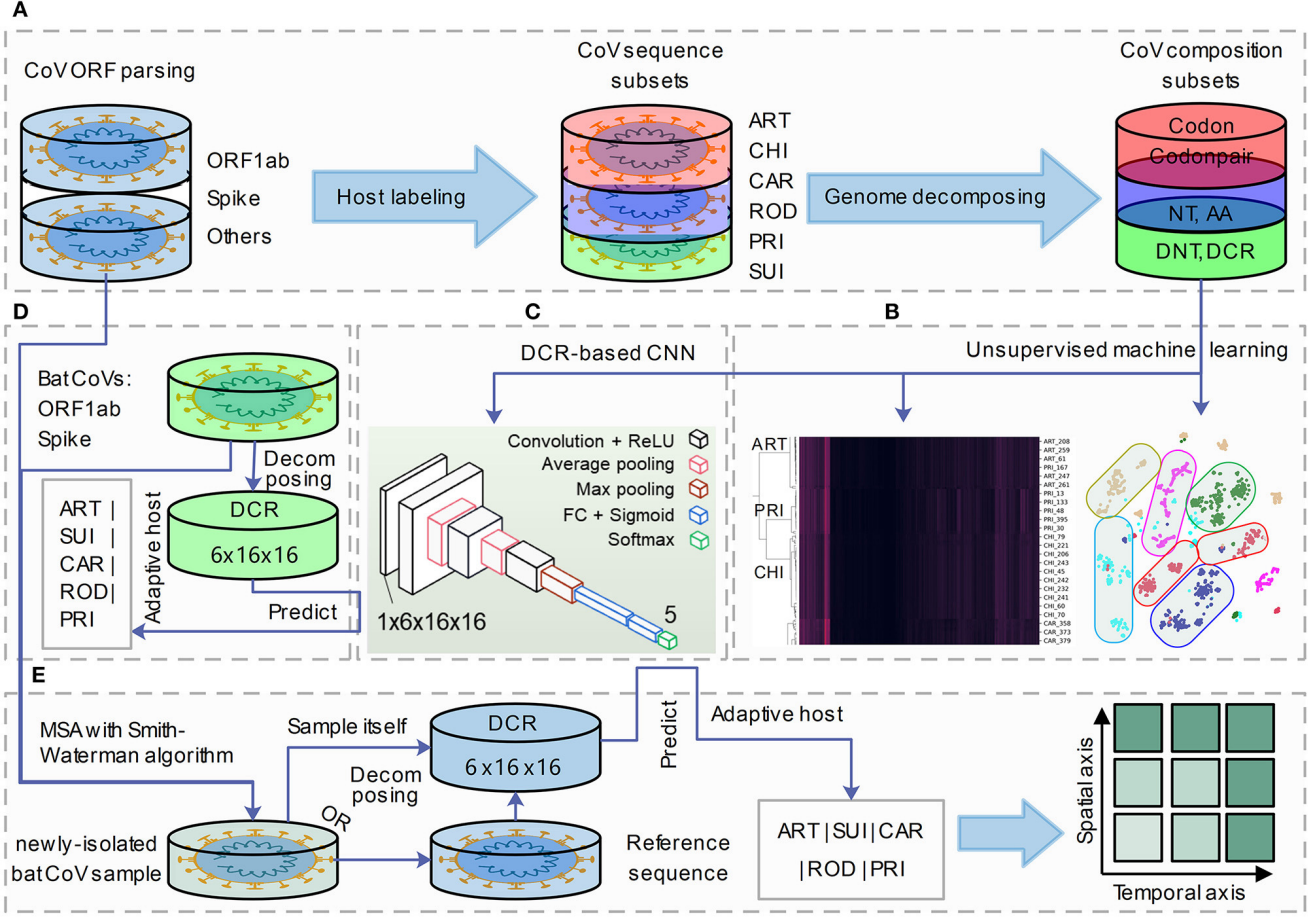
A pipeline for genome decomposition and adaptive host prediction of coronaviruses. The study was designed as the following pipeline of four sections. **(A)** Coronavirus (CoV) open reading frame (ORF) parsing (*ORF1ab* and *Spike*), adaptive host labeling (ART, Artiodactyla; CHI, Chiroptera; CAR, Carnivora; ROD, Rodents and Lagomorphs; PRI, Primates; SUI, Suiformes) and genome decomposition (into six types of NT, nucleotide; AA, amino acid; DNT, dinucleotide, codon; DCR, DNT compositional representation and codon pair); **(B, C)** unsupervised machine learning analysis (t-distributed stochastic neighbor embedding: t-SNE, Principal Component Analysis: PCA and hierarchical clustering) **(B)** and supervised deep learning (DCR-based Convolutional Neural Networks, CNN) prediction building of CoV genes; **(D)** field coronavirus sample collection, sequencing and Multiple Sequence Aligning (MSA); **(E)** host adaptation prediction of novel isolated CoV strains based on their decomposed DCR or based on their reference CoV sequences.

To represent viral genome composition, full-length CoV sequences were selected and labeled with each of the six adaptive hosts (ART, CHI, CAR, ROD, PRI, and SUI). Complete open reading frames (ORFs) of *Spike* and *ORF1ab* were parsed for adaptation analysis. Six types of codon-dependent compositional traits of mononucleotides (NTs, N_NTdimension_ = 12), amino acids (AAs, N_AAdimension_ = 20), DNTs (N_DNTdimension_ = 48), codons (N_codondimension_ = 64), DCR (N_DCRdimension_ = 1,536), and codon pairs (codonpairs, N_codonpairdimension_ = 3,721) were embedded for *Spike* and *ORF1ab*, respectively, with previously reported approaches (Li et al., 2022) (Figure 1A). Unsupervised machine learning methods such as t-SNE, PCA, and hierarchical clustering were performed to visualize the separation and clustering of CoVs based on their abovementioned traits (Figure 1B). A DCR-based CNN (Li et al., 2022) was utilized to classify the CoVs based on each of the five adaptive host labels (Figure 1C). Finally, the adaptive host was predicted for bat CoVs, which were recorded in the database (Figure 1D) or were newly isolated and sequenced CoV strains (Figure 1E).

### 3.2. Representation and visualization of DCR and other compositional traits for CoVs

**Figure 2:**
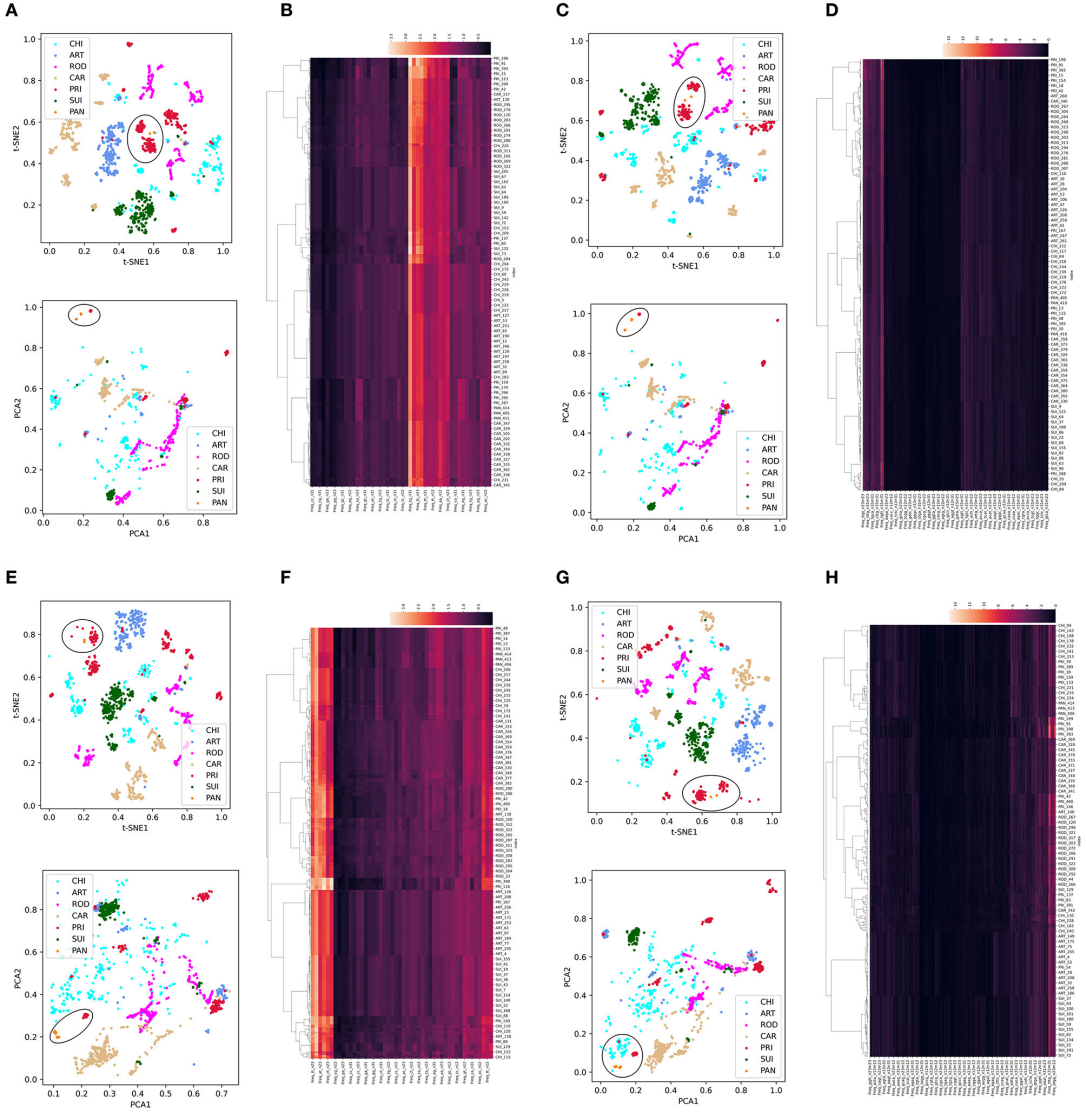
Distribution and clustering analysis of CoVs with host labels using unsupervised machine learning methods based on DNTs and DCR. Distribution of the two main components of compositional DNTs with t-SNE [**(A)** up] or PCA [**(A)** down] reduction and hierarchical clustering of 48 compositional DNTs of CoV *ORF1ab* **(B)**; **(C, D)** distribution of the two main t-SNE [**(C)** up] and PCA [**(C)** down] DCR components of *ORF1ab* and hierarchical clustering of all 1,536 DCR features **(D)**; **(E–H)** Similar unsupervised machine learning analysis of DNTs **(E, F)** and DCR features **(G, H)** of *Spike*.

Dimension reduction was performed using t-SNE or PCA into two main components for each trait type of the CoVs. We then used Synthetic Minority Over-sampling Technique (SMOTE) to correct the data imbalance among host labels by up- and down-sampling. Given the high importance of SARS-CoV-2-related pangolin CoVs, we also added pangolin CoV data for unsupervised learning analysis. In the ORF1ab DNT trait, we observed a clear separation among CoVs with the five host labels in the two reduced t-SNE components (upper part, Figure 2A) and a much more diffuse distribution in the two reduced PCA components (lower part, Figure 2A). The intra-host clustering and the inter-host separation were also indicated using the hierarchical clustering of *ORF1ab* DNTs (Figure 2B). Similar clustering and separation of *ORF1ab* DCR were also observed post-t-SNE/PCA reduction and using hierarchical clustering (Figures 2C, D). The *Spike in* DNT and DCR also indicated intra-host clustering and inter-host separation in both DNT and DCR traits using the three types of unsupervised machine learning methods (Figures 2E–H). Interestingly, the pangolin CoVs were closely clustered with PRI CoVs, either for the reduced DNT or DCR features of *ORF1ab* (Figures 2A–D) of *Spike*. Moreover, the compositional traits of AAs and NTs for *ORF1ab* (Supplementary Figures S1a–f) and *Spike* (Supplementary Figures S1g–l) and the compositional traits of codons and codonpairs for *ORF1ab* (Supplementary Figures S2a–f) and *Spike* (Supplementary Figures S2g–l) were also observed. Additionally, some obvious disseminated distribution for ROD or CAR samples was mainly enlarged for abnormally disseminated samples using SMOTE sampling; the wide distribution of CHI samples had no association with data sampling and probably implied the wide host adaptation of CHI CoVs. Taking these results together, there was a host specificity in DCR and other compositional traits for the *ORF1ab* and *Spike* of CoVs.

Additionally, the other three genes, *E, M*, and *N*, were analyzed for the abovementioned six types of compositional traits. The severe mixture was observed for each type of trait in the two-dimensional space of t-SNE1 and t-SNE2 or of PCA1 and PCA2 (in order of amino acid, NTS, DNTS, DCR, codons, and codonpairs, respectively, for a-f, Supplementary Figures S3–S5).

### 3.3. Performance of the DCR-based CNN model to predict adaptive hosts CoVs

**Figure 3:**
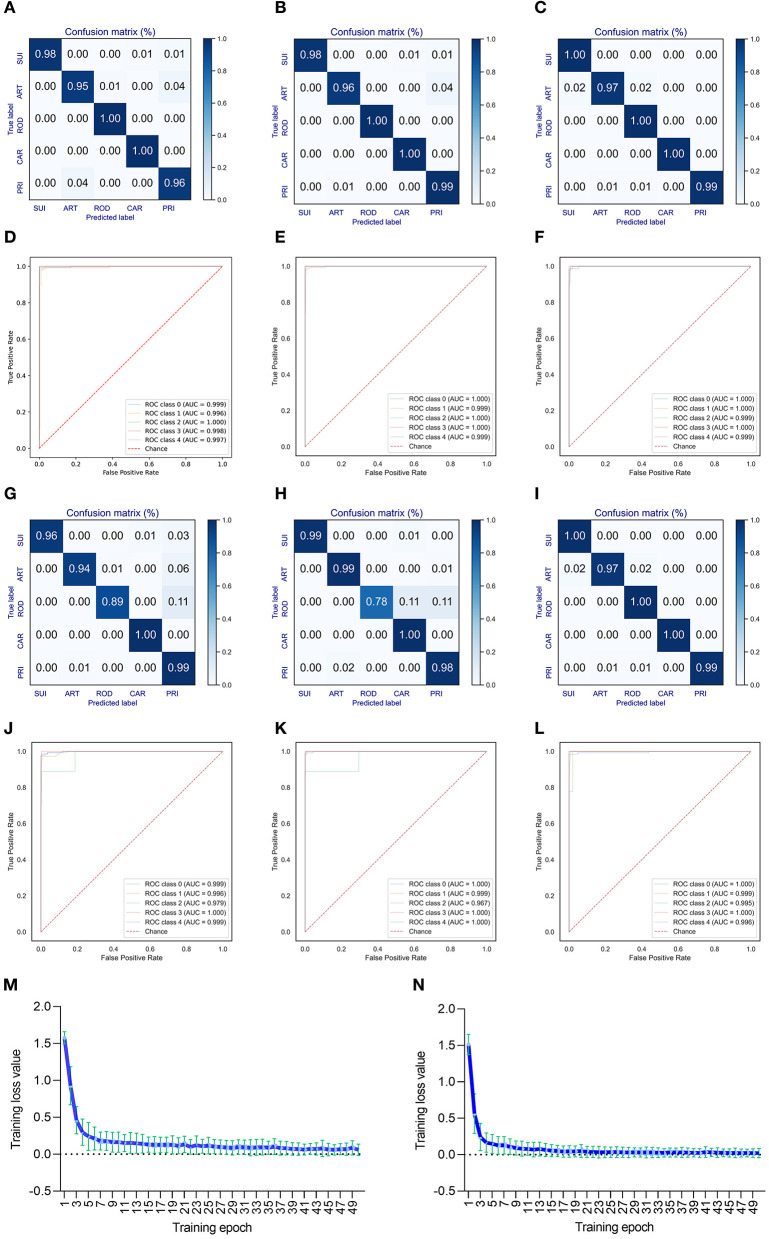
Performance of the DCR-based deep learning approach for predicting adaptive hosts of bat coronaviruses. The performance of the DCR-based deep learning predictor was evaluated with a confusion matrix [**(A–C)** for training epochs of 10, 30, and 50, respectively] and receiver operating characteristic ROC curve () [**(D–F)** for training epochs of 10, 30, and 50, respectively] for CoV *ORF1ab*; a similar evaluation was performed with a confusion matrix [**(G–I)**, respectively] and ROC [**(J–L)**, respectively] for CoV *Spike*. **(M, N)** Curve of the average training loss for validated data for the predictors for *ORF1ab* **(M)** and *Spike* **(N)**. ART, Artiodactyla; SUI, Suiformes; ROD, Rodents and Lagomorphs; CAR, Carnivora; PRI, Primates.

A deep learning model of CNN was built to predict the adaptation of bat CoVs to various types of mammalian hosts. The classification model with five labels (ART, CAR, ROD, PRI, and SUI) was trained using the 1,536-dimension DCR features of either *ORF1ab* or *Spike*. A training epoch-dependent performance elevation was observed for the classification of valid data based on DCR features of *ORF1ab* according to the confusion matrix (for epochs 10, 30, and 50, respectively, in Figures 3A–C; or for epochs 10–50, respectively, in Supplementary Figures S6a–e) or area under the receiver operating characteristic curve (ROC_AUC) (Figures 3D–F; Supplementary Figures S6f–j). Another model based on *spike* DCR features was also trained for the classification of CoV adaptive hosts. A high prediction accuracy was also obtained post-50-epoch training, as indicated by the confusion matrix (higher than 97% for epoch 50, Figures 3G–I; Supplementary Figures S6k–o) or ROC_AUC (Figures 3J–L; Supplementary Figures S6p–t). The training loss for either classifier descended quickly within the first 10 epochs and reached a plateau at approximately 20 epochs (respectively for *ORF1ab* and *Spike* Figures 3M, N; Supplementary Figures S6p–t).

**Figure 4:**
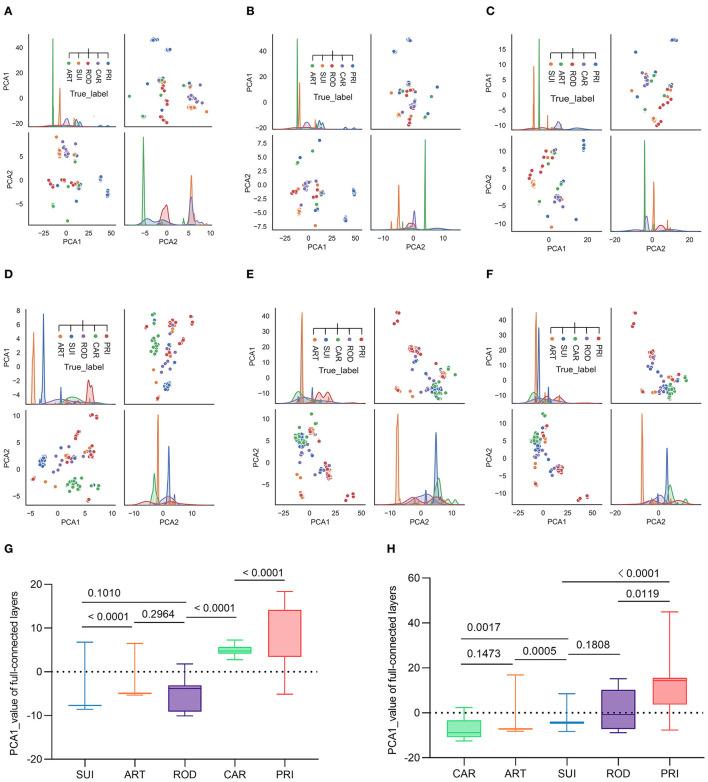
Visualization of the complete connection layer post-deep learning training of CoV *ORF1ab* and *Spike*. Full connection layers after three rounds of convolution were reduced by PCA into two principal components and plotted to visualize the distribution of CoV samples with the five host labels. Pair plots of reduced PCA1 and PCA2, post-training epochs of 10, 30, and 50, were plotted for *ORF1ab* **(A–C)** or *Spike* **(D–F)**, respectively. **(G, H)** Statistics of the reduced PCA1 value post-training epoch of 50 [**(G)** for *ORF1ab* and **(H)** for *Spike*].

To interpret the two trained classifiers, the reduction of the model's full-connected layer with PCA was visualized by plotting each pair of PCA1/PCA2 and PCA2/PCA1. The plotting results demonstrated that there was a sequential distribution of SUI, ART ROD, CAR, and PRI for the *ORF1ab* samples with five host labels for epochs 10, 30, and 50 (Figures 4A–C) or for 10–50 epochs (Supplementary Figures S7a–e). A significant separation of PRI (from other mammalian hosts) CoV samples was also observed from the distribution of the trained full-connected layer of *spike* DCR for epochs 10, 30, and 50 (Figures 4D–F) or for 10–50 epochs (Supplementary Figures S7f, g), with a different sequence of CAR, ART, SUI, and ROD for the other four host labels. The statistical analysis of the PCA1 values for each group indicated a significant difference between each neighboring pair of hosts in the *ORF1ab* samples (*P* < 0.01, except for ART vs. ROD with *P* > 0.5, Figure 4G). The difference was also significant for the neighboring ART/SUI or ROD/PRI *ORF1ab* samples (*P* < 0.01, Figure 4H).

### 3.4. DCR-based CNN predicts asymptotic bat-to-human adaptation of bat CoVs

**Figure 5:**
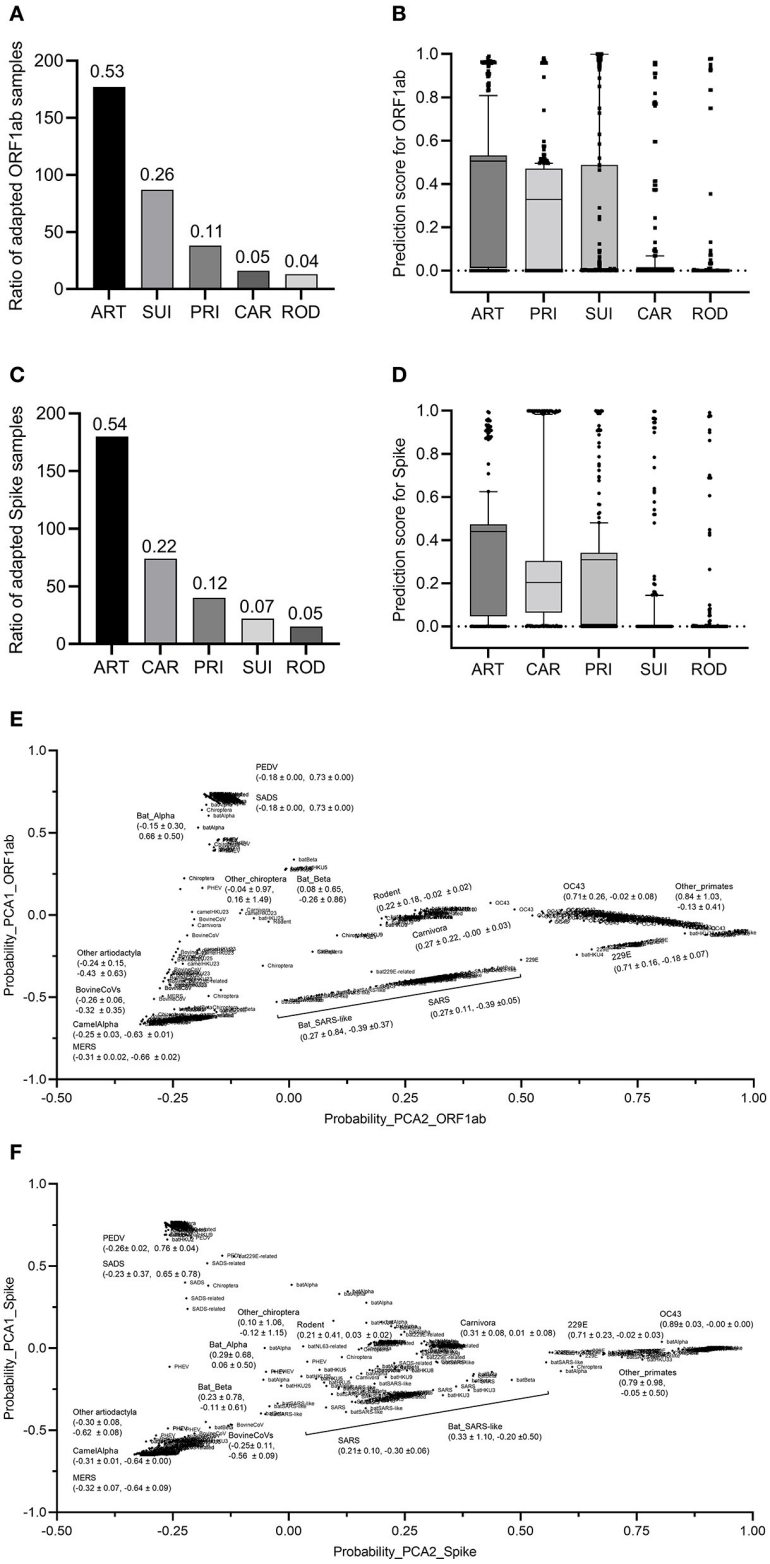
Prediction and prediction probabilities of bats and other coronaviruses. **(A, B)**: Prediction **(A)** and prediction probability **(B)** of *ORF1ab* for the adaptation to the five mammalian hosts of bat coronaviruses; **(C, D)** prediction **(C)** and prediction probability **(D)** of *Spike* for the adaptation to the five mammalian hosts of bat coronaviruses; **(E, F)** visualization of the PCA-reduced prediction probability of *ORF1ab* **(E)** and *Spike* **(F)** for bat and other coronaviruses.

To assess the adaptation of bat CoVs to other mammalian hosts, bat CoV sequences were fed to the two trained classifiers for *ORF1ab* and *Spike*. The results showed that 53% of CoV *ORF1ab* sequences were predicted as ART adaptive, while the percentages of adaptive samples for SUI, PRI, CAR, and ROD were 26, 11, 5, and 4%, respectively (Figure 5A). The average standardized probability of the predicted five groups of ART, PRI, SUI, CAR, and ROD were 0.640, 0.477, 0.276, 0.085, and 0.042, respectively (Figure 5B). The second classifier predicted almost the same percentage of Spike-adapted CV for ART hosts (54%). The percentages of adaptive samples for the other four types of hosts were 7, 12, 22, and 5%, respectively (Figure 5C), with an average standardized probability of 0.623, 0.451, 0.081, 0.456, and 0.048 for the five groups (Figure 5D). To further assess the distribution of bat CoVs and other mammalian CoVs in the adaptation space of mammalian hosts, five probability values for the five hosts were taken as a vector for each sample and were reduced to two main components with PCA. Interestingly, except for CoVs with an SUI host label, other mammalian but bat *ORF1ab* samples were almost linearly distributed, with ART samples on the lower left, CAR, and ROD samples in the middle, and PRI samples mainly on the upper right (Figure 5E), indicating a linear asymptotic adaptation shift from ART to CAR/ROD and then to PRI. Particularly, there was a linear-like distribution of all human CoV or human CoV-related ORF1ab samples in the two-dimensional space. MERS/bat MERS-related CoVs, SARS/bat SARS-like CoVs, and human CoVs of OC43, 229E, and others were successively distributed from the lower left to the upper right (Figure 5E). Similar linear asymptotic adaptation shifts of CoV *spike* samples were also observed (Figure 5F). Additionally, bat CoVs were disseminated in the adaptation space, with varied distances in PCA1 or PCA2 values for each of the five groups of CoVs (Figures 5E, F). Taken together, the two adaptation classifiers predicted a unanimous linear asymptotic adaptation shift from the ART host to humans.

## 4. Discussion

The present study aimed to predict the potential for viruses, such as influenza A viruses and coronaviruses, to cause infection and transmission in the human population.

Thus, we defined it as “the capability to infect humans easily, to transmit among populations efficiently, and to be virulent to some degree to humans” previously (Li et al., 2020, 2022). Genomic traits for virus adaptation have been biologically interpreted as shaping viral mRNA decay (Contu et al., 2021), methylation (Upadhyay et al., 2013), translation (Chen et al., 2020), replication efficiency (Forsberg, 2003; Bahir et al., 2009; Li et al., 2011), and antagonizing host anti-virus immune response (Xia, 2020), all of which reflect viral adaptive phenotypic traits to their hosts. Moreover, such adaptive genotypes were distinguishable and predictable with machine learning or deep learning approaches. Adaptation phenotypes of viruses to bats and other mammals are supported by parallel viral genotypes. A coarse-grained representation of the viral genome as compositional traits, such as DNT and DCR, is host-specific and predictable with machine learning or deep learning approaches for CoVs (Pollock et al., 2020; Li et al., 2022; Nan et al., 2022), influenza viruses (Taubenberger and Kash, 2010; Li et al., 2020), and other viruses (Bahir et al., 2009; Babayan et al., 2018; Chen et al., 2020). Fine-tuned sequential representation has been indicated to be sensitive to predicting the adaptation of SARS-CoV-2 Omicron sublineages with deep learning (Nan et al., 2022). In the present study, representative compositional traits of DCR and others confirmed the intra-host clustering and inter-host separability of various host-specific CoVs. Interestingly, there was a disseminated distribution of bat (CHI) CoVs into the areas of the CoVs with other host labels, indicating multiple adaptations to other hosts of bat CoVs. Additionally, the dispersed distribution of ROD samples was mainly caused by SMOTE up-sampling. Pangolin has been shown to play an intermediate role in the cross-species infection of SARS-CoV-2 viruses (Lam et al., 2020; Xiao et al., 2020) or MERS-CoV (Chen et al., 2020). The compositional traits indicated a close clustering of these pangolin CoVs with human CoVs, either for *ORF1ab* or *Spike* genes, implying a human adaptation. However, we did not set pangolin as an independent host label for supervised learning due to the small sample size of the whole genome and also due to the too-close clustering of pangolin viruses to human CoVs. Multiple genes other than *ORF1ab* and *Spike* might mediate the adaptation of CoVs to human and other mammalian hosts. However, the three other genes, *E, M*, and *N*, were mixed for CoVs of various host labels, suggesting less host specificity.

**Figure 6:**
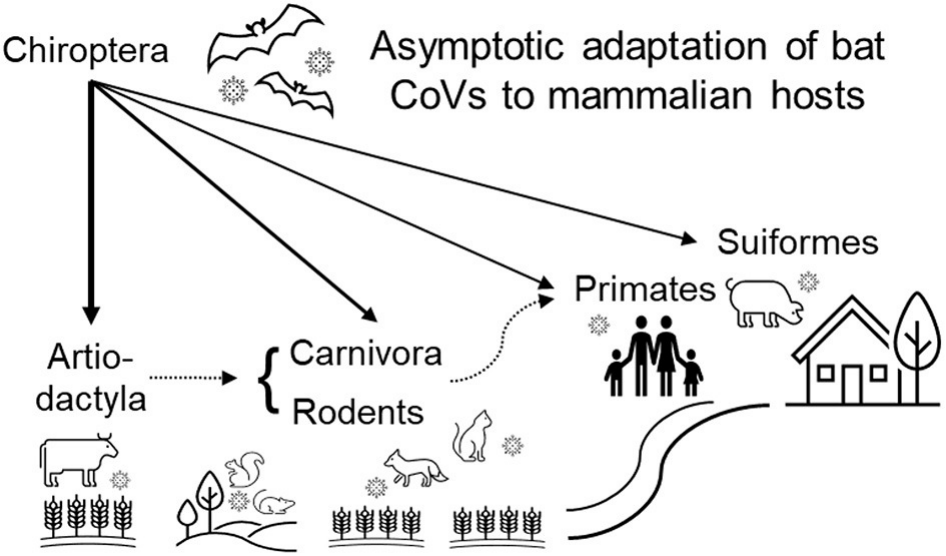
Summary diagram of adaptive hosts predicted by DCR-based deep learning. Adaption of Chiroptera CoVs to sequential ecological niches of ART/SUI, ROD/CAR, and then PRI are shown. ART, Artiodactyla; CAR, Carnivora; CHI, Chiroptera; PRI, Primates; ROD, Rodents and Lagomorphs; SUI, Suiformes.

In the present study, the deep learning classifier with five host labels (ART, CAR, ROD, SUI, and PRI) targeting either the *ORF1ab* or *Spike* gene, accurately predicted the host of the five groups of CoVs. A complete landscape of mammalian CoV samples in the predicted adaptation space constructed by the adaptation probability for the five hosts (Figure 5) unanimously showed a clearer intra-host clustering and inter-host separability of all CoV samples than the distribution of the original DCR features. Interestingly, a linear-like distribution of the CoV samples, except for the SUI CoVs, was observed in the adaptation space, suggesting CoV's asymptotic adaptation from ART to CAR/ROD and then to PRI hosts. Taking these results together, we proposed a possible niche distance-related landscape of host adaptation for bat CoVs (Figure 6): a dominant adaptation to the ART hosts, followed by a relatively less adaptation to CAR/ROD hosts, and finally to PRI hosts. Such asymptotic adaptation to the bat's close and far niche distances (Corman et al., 2018) reconfirmed the mediation of these natural hosts in the adaptation shift of bat CoVs to human beings. The ranked adaptation for bat CoVs provides more clues that CoVs might shift more probably from ART to a CAR/ROD host and then to humans than directly from CHI hosts, considering the closer niche distance between humans and these mediator hosts.

Additionally, the domestic pig in the SUI host type is the key mediator for the adaptation shift to a human host for the other major respiratory infectious agent, influenza A viruses (Neumann et al., 2009); however, the results in the present study indicated a significantly independent distribution of SUI CoVs from the linear-like and asymptotic distribution of the CoVs from other mammals. CoVs have been reported to cause infection and transmission in domestic pigs worldwide, such as porcine transmissible gastroenteritis virus (TGEV) (Brian and Baric, 2005), porcine enteric diarrhea virus (PEDV) (Lin et al., 2016), and swine acute diarrhea syndrome (SADS) CoV (Zhou et al., 2018). SUI CoVs are not likely to cause transmission in the human population, although the porcine delta coronavirus has been reported to infect malnourished Haitian children (Lednicky et al., 2021). SUI CoVs did not cause cross-species transmission in humans, as they were not closely related to human CoVs in the adaptation space predicted in this study. Therefore, we speculate that the risk of SUI CoVs threatening human populations is lower. However, it is important to note that overfitting can occur in machine learning or deep learning models to varying degrees. Additionally, there is a significant bias, with a smaller number of ROD CoVs and a much larger number of SUI or ART CoVs. The use of up-sampling for ROD CoVs and down-sampling for SUI and ART CoVs may lead to overfitting of the model and potentially explain the wide range of predicted adaptation probabilities (Figure 4).

In summary, the genomic dinucleotides represented as DCR indicate a host-specific separation and clustering that can predict a linear and asymptotic adaptation shift of bat CoVs from other mammals to humans through deep learning techniques.

## Data availability statement

The datasets presented in this study can be found in online repositories. The names of the repository/repositories and accession number(s) can be found in the article/Supplementary material.

## Author contributions

JL, J-FJ, TJ, and YT conceived the study. JL, FT, and SZ contributed to the acquisition and interpretation of data. S-SL, X-PK, J-QW, and WL performed data cleaning and statistical analysis. JL, SZ, ZL, Y-DL, and YF performed genome parsing and unsupervised and supervised learning with the assistance of J-FJ, TJ, and YT. JL drafted the manuscript, coded all scripts for genome parsing, deep learning, and data visualization. All authors contributed to the critical revision of the manuscript for important intellectual content.
