# Supplementary material for: Genomic representation predicts an asymptotic host adaptation of bat coronaviruses using deep learning
Source: Front Microbiol. 2023 May 5;14:1157608. doi: 10.3389/fmicb.2023.1157608 (PMC10198438; doi:10.3389/fmicb.2023.1157608)

**Supplementary Figure legends**

**Supplementary Fig. S1. Distribution and clustering analysis of CoVs based on amino acids or nucleotides post the dimension reduction with t-SNE or PCA.**

Distribution of the two main components with t-SNE (a) or PCA (b) reduction of 20 compositional amino acids features and hierarchical clustering of the 20 compositional amino acids of CoV *ORF1ab* (c); Distribution of two main t-SNE (d) and PCA (e) components and hierarchical clustering of 12 mono-nucleotides (NTs) of *ORF1ab* (f); g-l: Similar unsupervised machine learning analysis of amino acids (g-i) and NTs (k-l) of *Spike*.


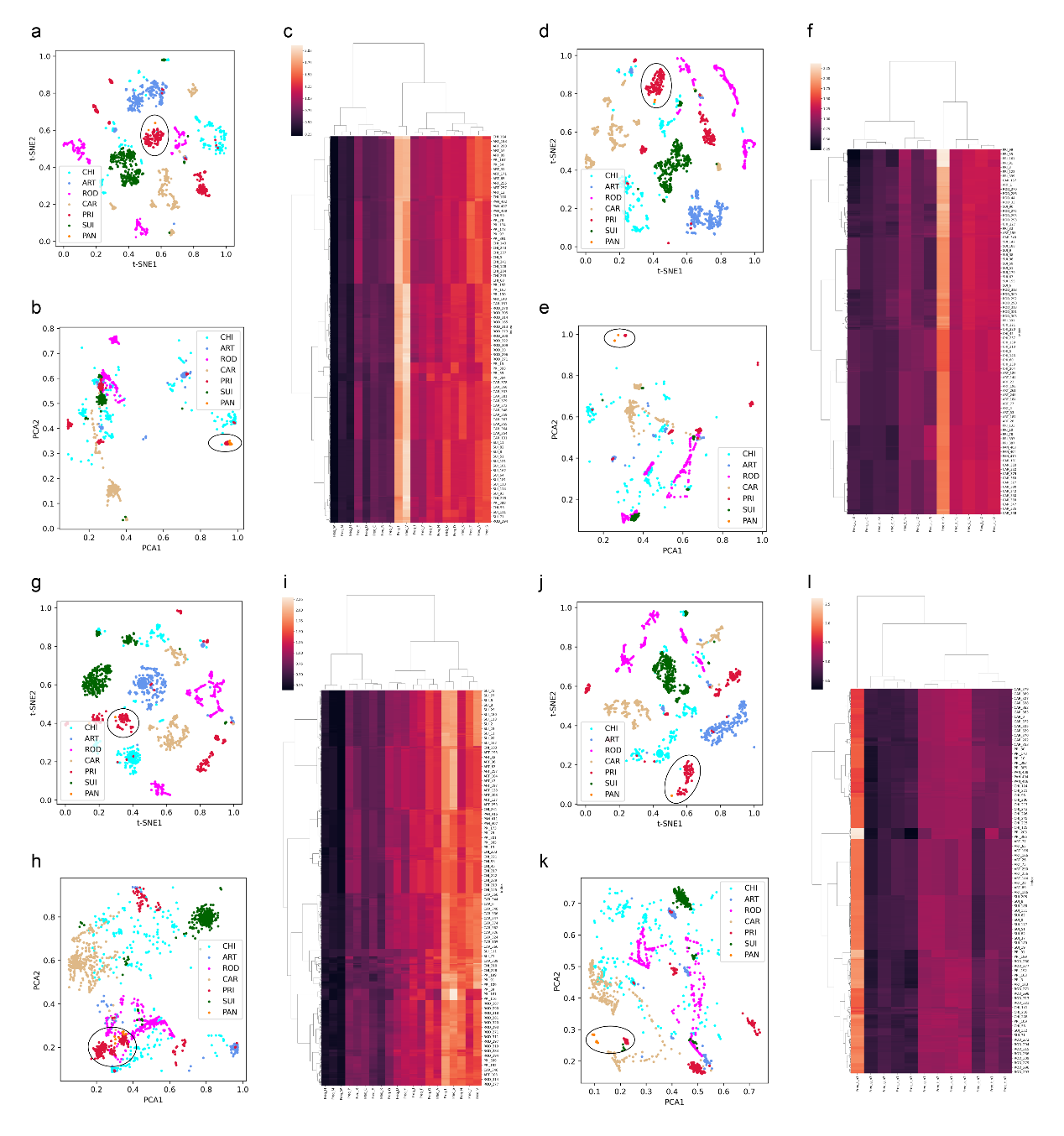


**Supplementary Fig. S2. Distribution and clustering analysis of CoVs based on codons and codonpairs post the dimension reduction with t-SNE or PCA.**

Distribution of the two main components of compositional NTs with t-SNE (a) or PCA (b) reduction and hierarchical clustering of 64 compositional codons of CoV *ORF1ab* (c); Distribution of two main t-SNE (d) and PCA (e) components and hierarchical clustering of all 3,721 codonpair features of *ORF1ab* (f); g-k: Similar unsupervised machine learning analysis of codons (g-i) and codonpair (k-l) of *Spike*.


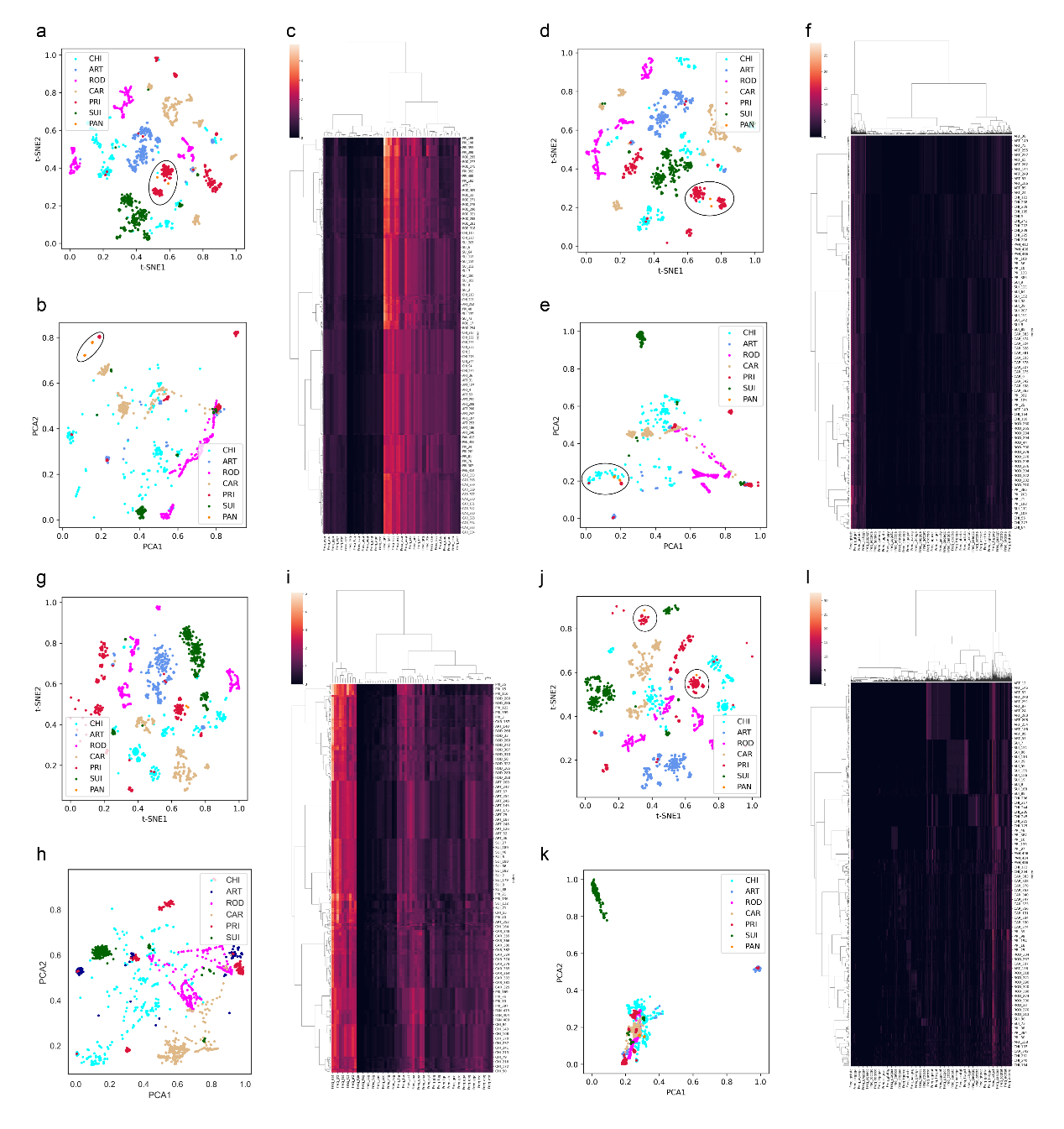


**Supplementary Fig. S3. Distribution and clustering analysis of CoV *E* based on compositional traits post the dimension reduction with t-SNE or PCA.**

Distribution of the two main components of t-SNE or PCA for 20 amino acids (a), 12 NTs (b), 48 DNTs (c), 1536 DCR (d), 64 codons (e) or 3721 codonpair (f).

**
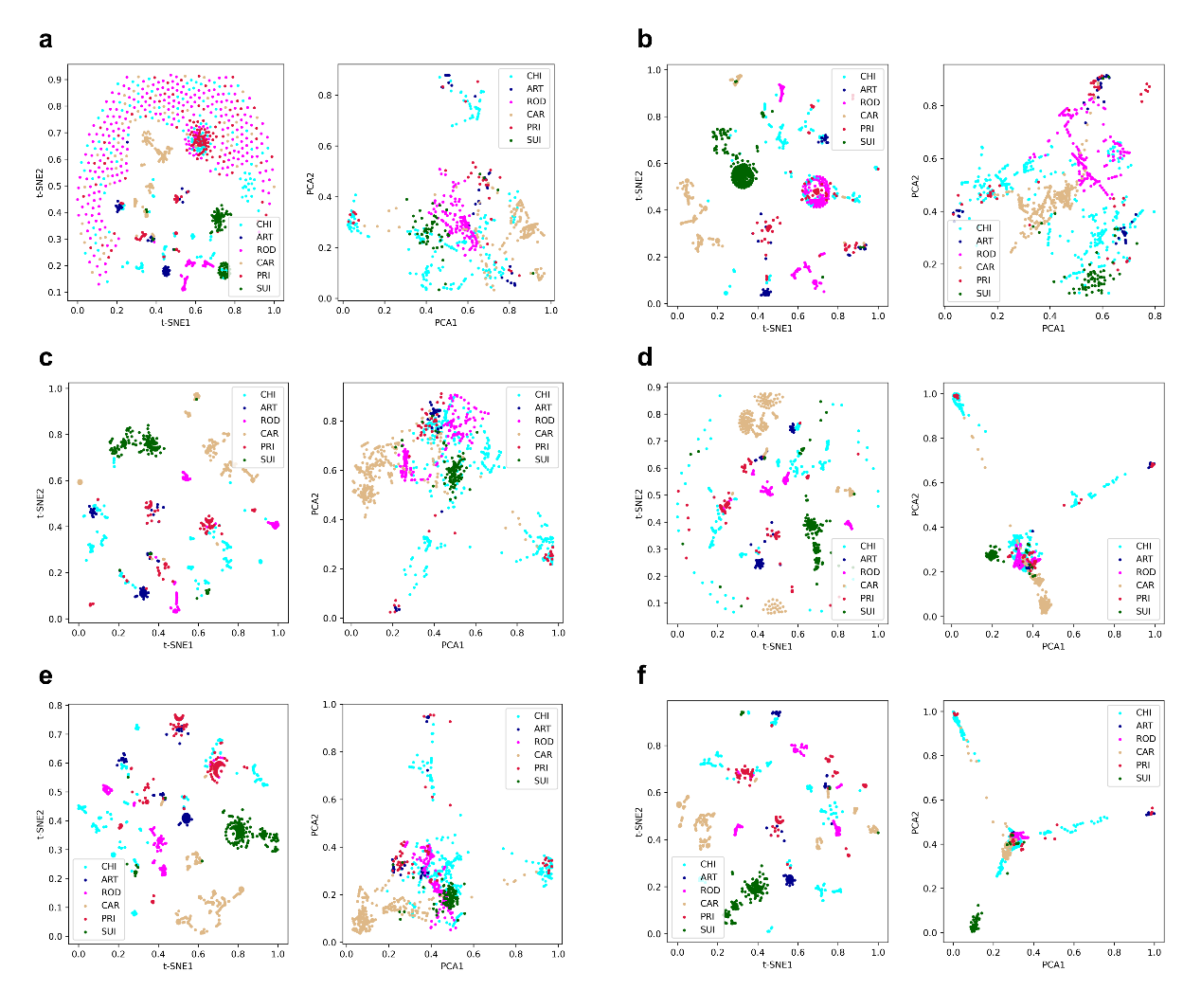
**

**Supplementary Fig. S4. Distribution and clustering analysis of CoV *M* based on compositional traits post the dimension reduction with t-SNE or PCA.**

Distribution of the two main components of t-SNE or PCA for 20 amino acids (a), 12 NTs (b), 48 DNTs (c), 1536 DCR (d), 64 codons (e) or 3721 codonpair (f).


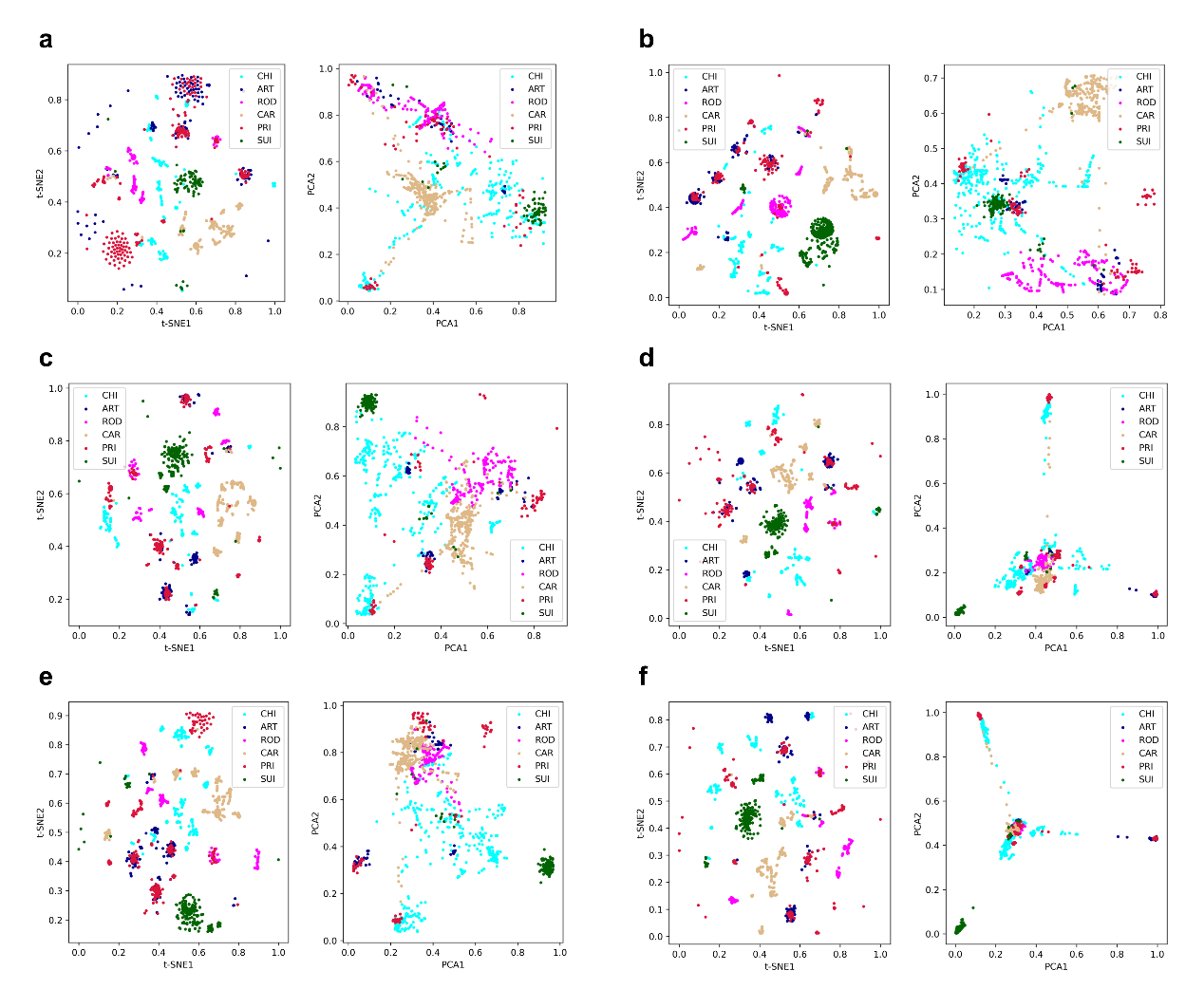


**Supplementary Fig. S5. Distribution and clustering analysis of CoV *N* based on compositional traits post the dimension reduction with t-SNE or PCA.**

Distribution of the two main components of t-SNE or PCA for 20 amino acids (a), 12 NTs (b), 48 DNTs (c), 1536 DCR (d), 64 codons (e) or 3721 codonpair (f).

**
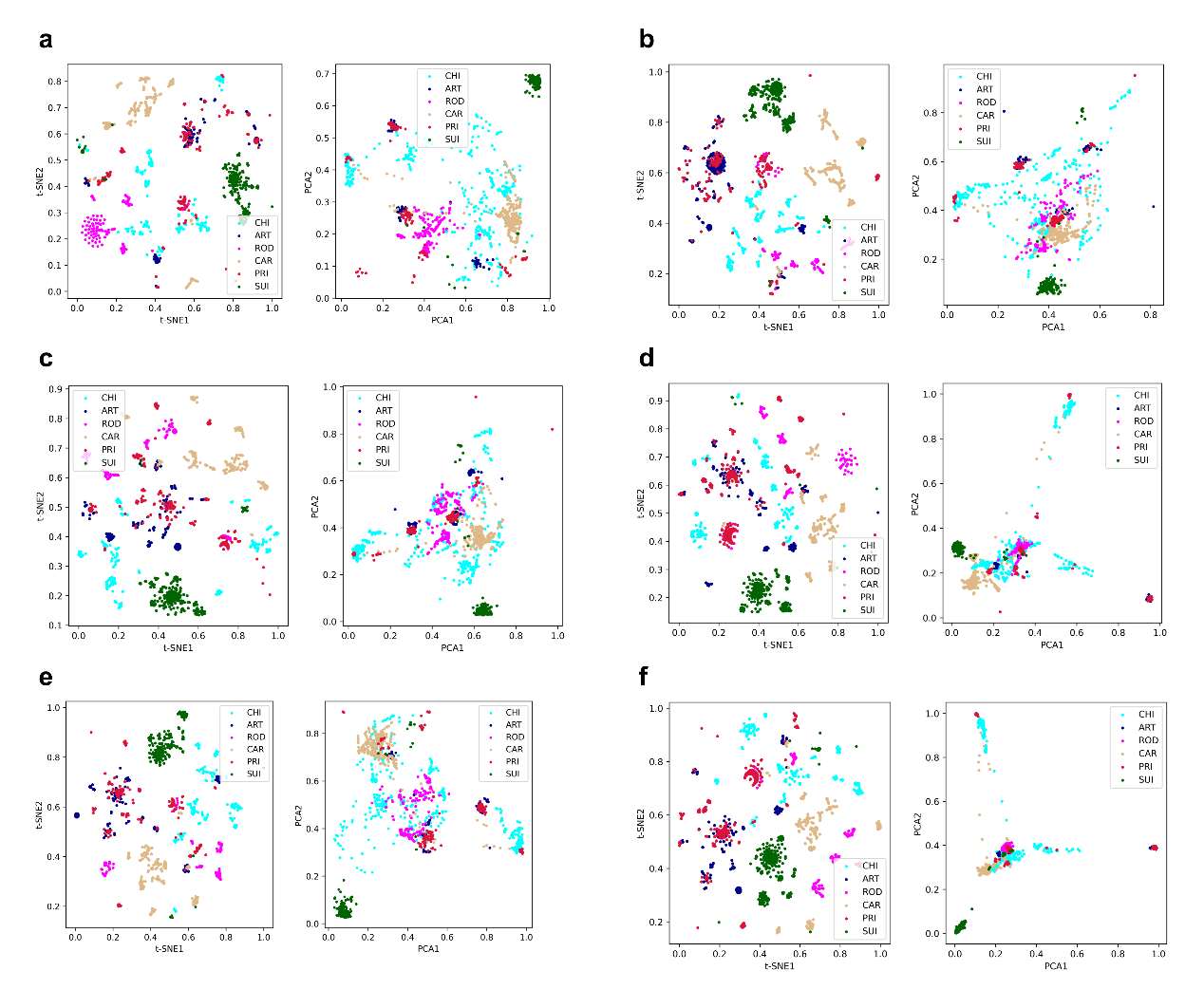
**

**Supplementary Fig. S6. Performance of a CNN predictor based on DCR for human adaptation prediction.**

The performance of the DCR-based CNN predictor was evaluated with Confusion matrix (**a-c** for training epochs of 10, 30 and 50 respectively) and receiver operating characteristic curve (ROC) (**d-f** for training epochs of 10, 30 and 50 respectively) for the CNN predictor for *ORF1ab*; Similar evaluation with confusion matrix (**g-i,** respectively) and ROC (**j-l,** respectively) for CoV *Spike*. **m** and **n**: Curving of average training loss for validate data for *ORF1ab* (**m**) and *Spike* (**n**). ART: Artiodactyla, SUI: Suiforms, ROD: Rodent_Lagomorpha, CAR: Carnivora, PRI: Primates.


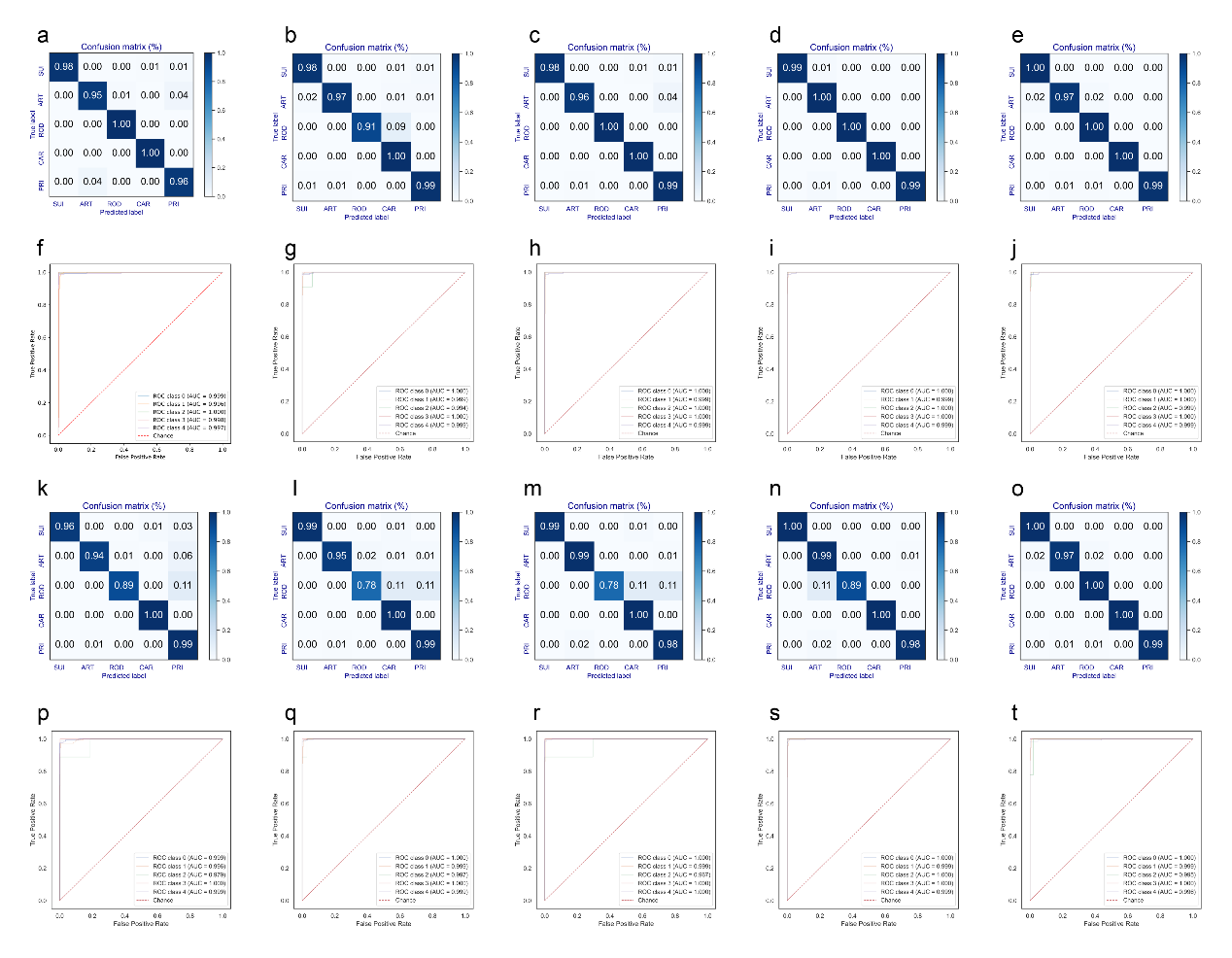


**Supplementary Fig. S7. Visualization of full connection layer post deep learning training of CoV *ORF1ab* and *Spike*.**

Full connection layers post three rounds of convolution were reduced by PCA into two main components and were plotted to visualize the distribution of CoV samples with the five host labels. Pairplot of the reduced PCA1 and PCA2, post training epochs of 10, 20, 30, 40 and 50 were respectively plotted for *ORF1ab* (**a-e**) or for *Spike* (**f-j**).


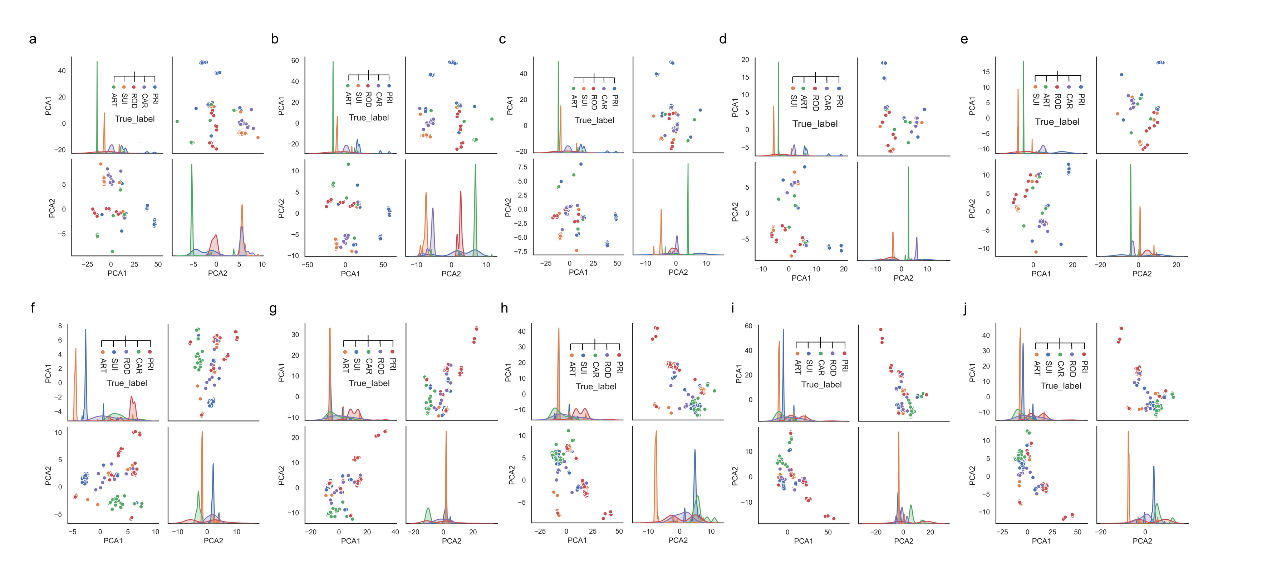

Supplement: Supplementary file 1 [file Data_Sheet_1.docx]
